# Mice lacking the Cβ subunit of PKA are resistant to angiotensin II-induced cardiac hypertrophy and dysfunction

**DOI:** 10.1186/1756-0500-3-307

**Published:** 2010-11-16

**Authors:** Linda C Enns, Kenneth L Bible, Mary J Emond, Warren C Ladiges

**Affiliations:** 1 Departments of Comparative Medicine, Physiology and Biophysics, and Biostatistics, University of Washington, Seattle, WA, 98195, USA

## Abstract

**Background:**

PKA is a ubiquitous, multi-subunit cellular kinase that regulates a number of different physiological responses in response to cAMP, including metabolism, cell division, and cardiac function. Numerous studies have implicated altered PKA signaling in cardiac dysfunction. Recently, it has been shown that mice lacking the catalytic β subunit of PKA (PKA Cβ) are protected from age-related problems such as weight gain and enlarged livers, and we hypothesized that these mice might also be resistant to cardiomyopathy.

**Findings:**

Angiotensin II (ang II) induced hypertension in both PKA Cβ null mice and their WT littermates. However, PKA Cβ null mice were resistant to a number of ang II-induced, cardiopathological effects observed in the WT mice, including hypertrophy, decreased diastolic performance, and enlarged left atria.

**Conclusion:**

The Cβ subunit of PKA plays an important role in angiotensin-induced cardiac dysfunction. The Cβ null mouse highlights the potential of the PKA Cβ subunit as a pharmaceutical target for hypertrophic cardiac disease.

## Background

PKA is a ubiquitous cellular kinase that is involved in regulating a vast number of different cellular processes. Several studies have implicated altered PKA signaling in cardiomyopathy [1,2]. For example, the onset of cardiac hypertrophy is influenced by alterations in muscle-specific A-kinase Anchoring Protein (mAKAP) signaling in myocytes. AKAPs subcellularly localize and modulate interactions between PKA and its downstream targets [3]. Deficiencies in the PKA pathway have also been linked both to cardiomyopathy in humans due to reduced phosphorylation of downstream targets such as cardiac troponin I [4], and to preservation of cardiac function against pressure overload in mice [5,6].

PKA is a tetrameric protein, consisting of two regulatory subunits and two catalytic subunits. Binding of cAMP to the regulatory subunits releases the catalytic subunits, which are then free to interact with and phosphorylate downstream targets. There are four isoforms of the regulatory subunit (RIα, RIβ, RIIα, RIIβ) and three types of catalytic subunits (Cα, Cβ, Cγ) [7,8]. C57/BL6J male mice lacking the regulatory RIIβ subunit have been found to be resistant to a number of age-related pathologies, including cardiac hypertrophy and decline [9]. We are currently studying mice lacking the PKA catalytic Cß subunit to establish whether they also enjoy age-delaying benefits. To date, we know that when challenged with a high fat, high calorie diet, these mice show robust obesity resistance, dramatic fat sparing effects in the liver, and protection against insulin resistance [10].

Cardiac hypertrophy is an increase in the mass of the heart in response to and to compensate for an increased workload. In the face of continued stress, hypertrophied diastolic and eventually systolic properties of the left ventricle become impaired, leading to decompensation and heart failure [11]. Angiotensin (ang) II is the effector of the renin-angiotensin system (RAS), and increases blood pressure by causing potent vasoconstriction through stimulation of angiotensin receptors in the vascular system [12]. We used ang II to administer a hypertensive challenge to the hearts of PKA Cβ null mice in order to establish whether or not they were protected against pressure overload-induced cardiac hypertrophy and dysfunction.

## Methods

PKA Cβ null mice lack expression of all PKA Cβ isoforms [13]. Mice were backcrossed to congenicity on a C57BL/6J background and genotypes were identified with PCR, both as previously described [10]. Seven each of 7 month-old, male PKA Cβ null mice and their WT littermates were surgically implanted with subcutaneous osmotic minipumps (model 1004; Alzet, Cupertino CA), to deliver a dosage of 0.7 mg/kg/day of Val^5^-angiotensin II (H1750; Bachem, Torrance CA) for 28 days. Blood pressures were measured before and after angiotensin treatment using the Coda-6 VPR tail cuff system (Kent Scientific, Torrington CT) [14,15] on conscious mice, as previously described, [16]. Non-invasive echocardiography and Doppler imaging were used before and 34 days after implantation of the minipumps to assess left ventricular mass, left atrial size, the velocity of the mitral valve annulus, isovolumic contraction and relaxation and ejection times, and the ratio of early to late diastolic filling (Ea/Aa). At the end of the experiment, ang II-treated mice were euthanized and their hearts weighed and compared with heart weights from another cohort of similarly aged, unchallenged mice. Probabilities of difference between groups were calculated using the Student's T-test; P's < 0.05 were considered to be statistically significant and are included in figures. All protocols were approved by the University of Washington Institutional Animal Care and Use Committee.

## Results

### PKA Cβ null mice are resistant to angiotensin-induced cardiac hypertrophy and dysfunction

**Figure 1:**
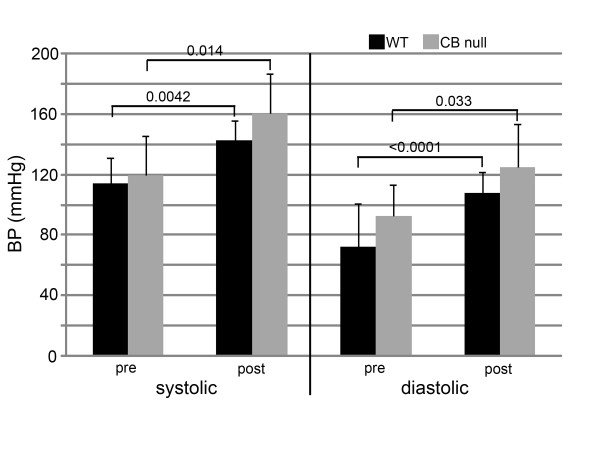
**Angiotensin II causes hypertension in Cβ null mice and WT littermates**. Blood pressure measurements from Cβ null mice and their WT littermates, pre and post treatment with angiotensin II. Both genotypes showed similar systolic and diastolic blood pressure before angiotensin treatment. Following 4 weeks of treatment, both Cβ null and WT mice showed similar and significant increases in systolic and diastolic blood pressure. n = 7 mice per genotype; error bars indicate standard deviations. Probabilities < 0.05 indicated on graph.

**Figure 2:**
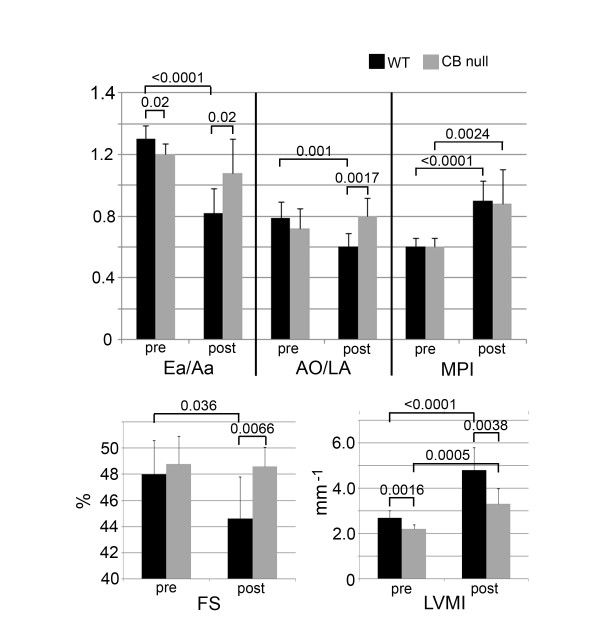
**PKA Cβ null mice are resistant to angiotensin II-induced cardiac hypertrophy and dysfunction**. Echocardiography and Doppler analyses of WT and PKA Cβ null littermates, pre and post angiotensin treatment. 5 parameters of cardiac morphology and function were measured: FS (fractional shortening of the left ventricle), Ea/Aa (ratio of early over late diastolic filling), AO/LA (ratio of the aortic diameter/left atrial diameter), LVMI (left ventricular mass index, standardized to tibia length) and MPI (mass performance index). The hearts of angiotensin-treated PKA Cβ null mice were significantly superior to those of WT littermates, for 4 of the 5 parameters measured. n = 7 per genotype; error bars represent standard deviations. Probabilities < 0.05 indicated on graph.

**Figure 3:**
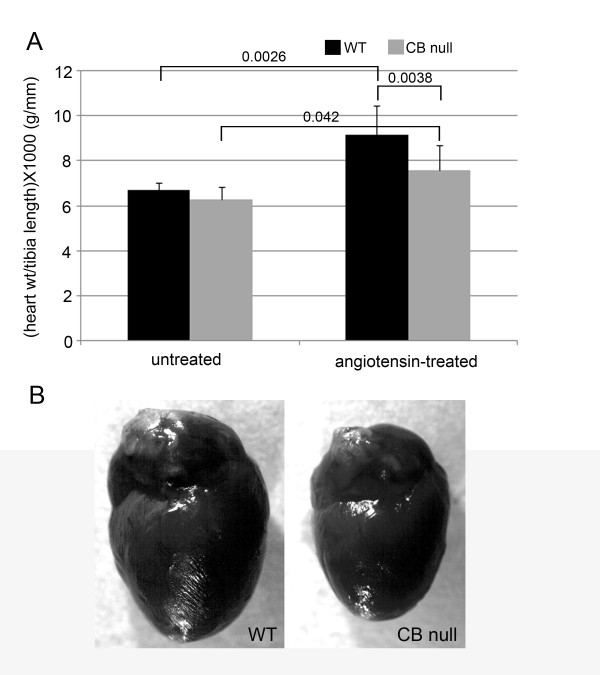
**Hearts from PKA Cβ null mice are resistant to hypertrophic effects of angiotensin**. **A**. Heart weights of WT and PKA Cβ null mice, before and after treatment with angiotensin II. Hearts of unchallenged mice showed similar weights regardless of genotype, but hearts of WT mice after angiotensin II treatment were significantly larger that those of PKA Cβ null littermates. Heart weights standardized to tibia length. n = 7 per genotype; error bars represent standard deviations. Probabilities < 0.05 indicated on graph. **B**. Hearts of angiotensin II-treated WT mice were visibly larger than those of Cβ null littermates. Echocardiographical measurements of LVMI were confirmed both visually and by weighing the hearts; the angiotensin II-treated WT heart on the left was found using echocardiography to have an LVMI of 4.07 while the challenged Cβ null heart on the right was found to have an LVMI of only 2.15.

Previous experiments have shown that insertion of subcutaneous saline pumps into mice, using our method does not produce any cardiovascular effects (data not shown). Four weeks of treatment with pumps containing angiotensin II caused a systolic and diastolic blood pressure increase of 25 and 50%, and 35 and 36%, for WT and PKA Cβ null mice, respectively (figure 1). Significant differences in blood pressure were not found between genotypes, either before or after ang II treatment. Echocardiography and Doppler imaging show similar cardiac performance between unchallenged, 7 month-old PKA Cβ null mice and their WT littermates (data not shown); however, when challenged with angiotensin II, compared to WT, mutants were found to be resistant to cardiac dysfunction in 4 of the 5 parameters measured (figure 2). Mutants displayed only a 60% increase in left ventricular mass index compared to over 100% in the WT. WT mice also showed significant decreases in fractional shortening of the left vetricle, compared to their mutant littermates which showed no decreases at all. The left atrium of the WT hearts, showed, on average, a significantly larger increase in size in response to ang II, reflected by a 25% decrease in the average aorta/left atrium ratio (AO/LA) compared to no change in the mutants. The ratio of early to late diastolic filling (Ea/Aa) decreased by over 40% in WT compared to about 10% in mutants, indicating significantly worse diastolic dysfunction. The only parameter equally affected in both genotypes was mass performance index (MPI). Results from echocardiography showing differences between genotypes in ang II-induced hypertrophy were confirmed upon euthanization of the mice. Unchallenged PKA Cβ null and WT mice had similar heart weights of about 0.15 g. After 28 days of ang II treatment, however, the hearts of WT mice were 38% larger, while PKA Cβ null hearts showed a significantly smaller increase of only about 17% (figure 3A). The difference in heart size between genotypes of ang II treated mice was clearly visible (figure 3B).

## Discussion

We show that disruption of the PKA catalytic subunit Cβ protects mice from angiotensin II-induced cardiac hypertrophy and dysfunction. In this study, a low dosage of angiotensin II (ang II) was used to effectively induce hypertension in WT, C57BL/6J mice and their PKA Cβ null littermates. After being challenged for 4 weeks with ang II, both genotypes showed a similar hypertensive response. In spite of similar systolic and diastolic blood pressure increases in response to ang II compared to WT, Cβ null mutants displayed smaller hearts and improved cardiac function in 4 of 5 echocardiographical parameters measured including left ventricular mass index, fractional shortening, ratio of early to late diastolic filling, and ratio of aortic to left atrial diameter. Only mass performance index showed no difference between genotypes. The role that ang II plays in the renin-angiotensin system (RAS) is known to be pivotal in the regulation of blood pressure [17]. Resistance of PKA Cβ null mice to cardiac hypertrophy demonstrates that PKA plays a role in the mediation of hypertension and its myopathological effects, although what that role is remains to be elucidated.

It has been known for some time that the β-adrenergic (β-AR)/adenylyl cyclase/PKA pathway, which is central to stimulating cardiac function, is dysfunctional in heart failure [18]. That β-AR signaling is detrimental to cardiac function is supported by clinical studies in humans showing that blockade of β-AR receptors improves survival in heart failure patients [19], and by studies on transgenic mice, showing that chronic activation of the cAMP-PKA pathway by cardiac-specific overexpression of β-AR, Gsα, and the α-catalytic subunit of PKA result in cardiomyopathy [2,20]. Disruption of adenylyl cyclase 5, which was shown to diminish cAMP-PKA signaling in the heart by 30-40%, was also shown to protect the murine heart from pressure overload-induced decompensation, although it did not affect the development of hypertrophy [5]. β-AR signaling can be overstimulated by hypertension [21], and PKA is known to instigate cardiac hypertrophy in response to elevation of cAMP by β-adrenergic agonists [22]. A reduction in the response of the β-AR pathway to hypertension could be the reason for the protective cardiac effects of the Cβ null mutation. There are, however, opposing studies that point to a protective role for the β-AR/AC/PKA pathway in response to hemodynamic overload. In humans, PKA-dependent phosphorylation of cardiac troponin I (TnI) has been found to be reduced in dilated cardiomyopathy [4], supporting the idea that loss of responsiveness of the β-AR pathway plays a role in cardiomyopathy. This finding is also supported in mice: overexpression of two types of adenylyl cyclase in the heart result in improved cardiac function [23,24]. Another PKA mutant mouse model lacking the regulatory RIIβ subunit of PKA [25,26], displays an obesity resistant phenotype similar to the Cβ null mutant [9], and is thought to be sensitive to β-adrenergic activation [27,28], an idea that is supported by their exaggerated sensitization response to amphetamine [29]. Similarities in other phenotypes between RIIβ null and Cβ null mutants indicate that they may also share enhanced β-AR signaling. It is unknown why in some cases, loss of β-AR signaling seems protective to cardiac function, and in other cases, the opposite seems to be true. The conflicting data does indicate that disruption of the different components of the β-AR pathway, and even different components of the PKA enzyme itself, have different consequences on cardiac performance. It is also unknown if and how the β-AR pathway is affected in PKA Cβ null mice, but the idea that alterations in this pathway may be in part responsible for the protective effects of the Cβ mutation on ang II-induced cardiomyopathy needs to be taken into consideration.

Other potential roles for PKA in protection against cardiac hypertrophy and dysfunction are numerous and diverse. Cyclic AMP signaling regulates a vast number of cellular processes, including cellular growth [30]. Specifically, activation of cAMP-PKA signaling has been shown to inhibit smooth muscle proliferation [31]. Like angiotensin II, cAMP/PKA transiently stimulates the expression of immediate-early genes [32]. In addition, PKA is known to regulate activity of some of the same pathways both activated by ang II and linked to cardiac hypertrophy; for example, PKA modulates ANF-dependent cGMP accumulation in renal cells [33]. Hypertrophy of cardiomyocytes in response to hypertension is thought to compensate for wall stress, and is characterized by an increase in cell size and enhanced protein synthesis [34]. Stretching of cardiomyocytes in response to haemodynamic overload is known to increase protein synthesis by activating second messengers such as Raf-1 kinase and extracellular signal-regulated protein kinases (ERKs) through activation of protein kinase C (PKC) [35]. Activation of PKA has been shown to have a synergistic effect on PKC-induced stimulation of Raf-1 and MAP kinases in rat cardiomyocytes[36,37], and disruption of PKA Cβ may reduce this effect. Growth factors may also play a role. For example, epidermal growth factor receptor (EGFR) phosphorylation is known to be involved in the development of pressure overload-induced cardiac hypertrophy [38]. In liver, disruption of Cβ leads to a reduction in EGFR levels [39], although this result still needs to be confirmed in the heart. Other downstream targets of PKA in myocytes include the L-type Ca2^+ ^channel in the sarcolemma, the ryanodine receptor (RyR2), and phospholamban in the sarcoplasmic reticulum (SR) [40,20]. There is substantial evidence that calcium signaling pathways play a role in cardiac hypertrophy [41,42], supported by the finding that its development in rats, in the presence of hypertension can be inhibited by blockade of L-type calcium channels [43]. Finally, PKA has recently been found to inhibit nuclear export of histone deacetylase 5 (HDAC5), resulting in inhibited gene transcription and attenuated phenylephrine and angiotensin II-induced rat cardiomyocyte hypertrophy [44]. HDACs play a role in the transcriptional regulation of myocyte enhancer factor 2 (MEF2), a transcription factor that activates many cardiac genes and is known to be involved in the development of cardiac hypertrophy [45].

## Conclusions

This study shows a clear role for the β catalytic subunit of PKA in ang II-induced cardiomyopathy. Not only does it illustrate the usefulness of the PKA Cβ null mouse for the study of the role of PKA signaling in heart disease; it also highlights the potential of the PKA Cβ subunit as a pharmaeutical target in the treatment of cardiac hypertrophy and dysfunction.

## Competing interests

The authors declare that they have no competing interests.

## Authors' contributions

WCL conceived the study. KLB performed the echocardiography and Doppler analyses. LCE was responsible for the study's design and coordination, performed the statistical analyses of the data, and drafted the manuscript. All authors read and approved the final manuscript.
